# Thermosensitive Interfacial Migration of 5-FU in the Microenvironment of Pluronic Block Copolymers

**DOI:** 10.3390/polym13162705

**Published:** 2021-08-13

**Authors:** Tz-Feng Lin, Shih-Hsuan Yeh

**Affiliations:** 1 Department of Fiber and Composite Materials, Feng Chia University, Taichung 407, Taiwan; 2 Master’s Program of Electrical and Communications Engineering, Feng Chia University, Taichung 407, Taiwan; a0912939549@gmail.com

**Keywords:** anticancer drug, 5-fluorouracil, drug release, Pluronic block copolymer, microenvironment

## Abstract

Chemotherapy is one of the most important ways to treat cancer. At present, chemotherapy medicines are mainly administered by intravenous injection or oral administration. However, systemic medical care requires the dosage of high concentrations of drugs to defeat the malignant tumor growth. In recent years, the use of polymer composites for local and sustained drug release has become an important field of research to minimize side effects due to high-concentration chemotherapy drugs. Here, ^19^F-{^1^H} heteronuclear Overhauser enhancement spectroscopy (HOESY) was used to study the micellular environment of the F-containing chemotherapeutic drug 5-FU in Pluronic F127, Pluronic L121, and F127/L121 binary blending composites. The distribution of 5-FU in micelles is related to the PEO and PPO segment length of Pluronic polymers and the environmental temperature. The drug release tests further confirm that if 5-FU medicines were loaded in the PPO segment inside the micelles, the purpose of the prolonged drug release carrier is achieved.

## 1. Introduction

The fluoropyrimidine 5-fluorouracil (5-FU) is an antimetabolite drug that is widely used for cancer treatment in the abdominal cavity [1]. Increased understanding of the mechanism of 5-FU has led to the development of its anticancer activity. However, 5-FU has a chemical structure similar to uracil and thymine and interferes with nucleotide synthesis and incorporates into DNA, which may have a mutational impact on both surviving tumor and healthy cells [2]. Additionally, 5-FU could trigger cancer cell-initiated anti-tumor immunity to reduce the tumor burden, and to improve therapeutic effectiveness for colon and other cancers [3]. Despite these advances, drug resistance remains a significant limitation to the clinical use of 5-FU [4]. To design a comprehensive and effective 5-FU drug delivery system for targeting tumors would be a part of precision medicine.

The poly(ethylene oxide)-poly(propylene oxide)-poly(ethylene oxide) block copolymer (PEO-PPO-PEO) (commercially available as Pluronics and Poloxamers) is an amphiphilic block copolymer that can self-assembly into micelles in aqueous solution. Enormous study shown that the micellular structure is a practical approach for incorporating medicines, as it not only builds a drug delivery system but also applies to gene and cancer therapies [5,6,7,8,9,10]. The key attribute for the biological activity of Pluronic block copolymers is their ability to interact with multidrug-resistant cancer tumors with respect to various anticancer agents both in vitro and in vivo [11,12,13].

Studies of Pluronic F127 have been carried out in transparent flowing solution at ambient temperature, but it became a hydrogel at body temperatures above 30 °C [14]. This Pluronic F127 sol-gel transformation with 5-FU loading is able to effectively induce the death of gastric cancer cells using a hollow microneedle injection [15]. Interactions between non-ionic micelles (not limited to Pluronic block copolymers) and small molecules (not limited to 5-FU) can be found in the literature too [16,17]. Further, binary mixing of Pluronic F127 and Pluronic L121 produces tiny micelles that satisfy the stability criteria by demonstrating appropriate thermodynamic and kinetic stability in biologically relevant media as pharmaceutical ingredients [18].

Here, the characterization of 5-FU in Pluronic F127, Pluronic L121, and their binary mixing was studied by ^19^F-{^1^H} heteronuclear Overhauser enhancement spectroscopy (HOESY). When the fluorine-19 nuclei were irradiated on 5-FU in the NMR, the identified polarization of hydrogen-1 nuclei could only be detected in the range of 6 Å from the fluorine-19 nuclei. The utilization of dipolar coupling (i.e., cross-relaxation) presents the distribution of 5-FU medicine loading and its interfacial movement in the Pluronic micelles. The main results can significantly increase the understanding of drug delivery systems in prolonged drug delivery. 5-FU/F127/L121 Pluronic blending composites provide insights into the design of future drug delivery systems, resulting in major progress in tumor therapy. Additionally, the cellular uptake or expulsion of incorporated chemotherapeutic drugs in Pluronic micelles mimics the normal cell function as a microenvironment.

## 2. Materials and Methods

Tween 20, Dimethyl sulfoxide (DMSO), Deuterium oxide (D_2_O) with 99.9 atom % D, and Pluronic^®^ polymers of F127 and L121 were purchased from Sigma-Aldrich and used as received without any purification. Pluronic^®^ polymers of F127 and L121 have an average Mn at ~12,600 g/mol and ~4400 g/mol, respectively. Both Pluronic F127 and Pluronic L121 were diluted with D_2_O, respectively, until 4.2 wt% and 50 wt%. 5-FU medicine was purchased from AdooQ^®^ Bioscience and stored in DMSO as 0.2 M at 4 °C. For spectra experiments, the as-prepared samples were well mixed by an ultrasonic processor and then kept in Eppendorf tubes before usage or transfer. Every control sample had the same composition as its relative experimental sample. Briefly, the total volume of every sample was taken in 600 μL, and the binary mixing of Pluronic^®^ polymers was proceeded by the ratio of volume.

Based on the ^1^H and ^19^F NMR characterization, because 5-FU has a fluorine atom, this feature allowed us to determine the radio frequency through chemical shift coupling and the corresponding environment. Accordingly, the chemotherapeutic drug 5-FU was used as an indicator to observe its distribution between the interface of F127, L121, and their Pluronic blending composites. All NMR experiments were performed on a JEOL ECZ400S/L1 spectrometer with an HFX probe at a resonance frequency of 399.78 MHz for ^1^H and 376.17 MHz for ^19^F. The 1D selective ^19^F-{^1^H} heteronuclear Overhauser enhancement experiments were performed using the pulse program (^19^F_^1^H_1d_selhoesy) from the JEOL library. The scan numbers were set at 64, with a relaxation delay of 3 s. Additionally, a mixing time of 2.7 s was used. The FID results were treated by the software of delta v.5.3.1.

To measure the amounts of 5-FU medicine during drug release experiments, 600 μL Pluronic blending composites with or without 5-FU was placed in triplicate in an Eppendorf tube, and 600 μL PBS (pH 7.4, containing 0.1% Tween 20 (*v*/*v*)) was pipetted onto the upper part of the Eppendorf tube. The as-prepared Eppendorf tube was stored in a 37 °C sink condition under agitation at 100 rpm for the release monitoring of 5-FU. In each time set of the measurement, the amount of drug-released 5-FU was determined at OD265 by using a calibration curve generated from known concentrations of 5-FU. Average was taken from three recorded 5-FU concentrations with respect to the time evolution. Fresh PBS was replaced each time for further analysis.

## 3. Results and Discussion

**Figure 1:**
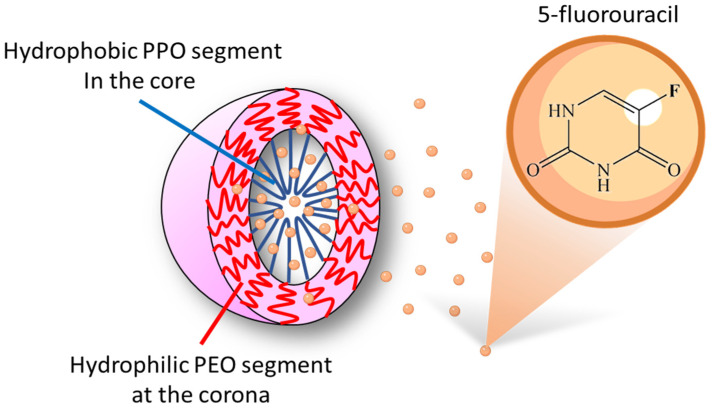
Schematic representation of the chemical structure of 5-Fluorouracil (5-FU). 5-FU medicine was loaded in the hydrophobic core of Pluronic micelles.

5-FU is a common chemotherapeutic medicine for intravenous injection or oral administration [19]. The chemical structure of 5-FU is depicted in Figure 1. Pluronic^®^ polymers were self-assembled into Pluronic micelles with a hydrophilic PEO segment at the corona and a hydrophobic PPO segment in the core. Studies elucidated that 5-FU would be hydrophilic [20,21,22,23,24] or hydrophobic molecules [25,26,27,28]. However, for drug release, the encapsulation of the drug in micelles was highly dependent on its loading location rather than the intrinsic solubility of the drug in aqueous solution. Therefore, the important thing is how to extend the effective time of drug release from a designated polymeric area. This study used NMR technology to identify the position of the 5-FU medicine in the drug release carrier, trying to propose a mechanism simulating the microenvironment of the cell operation.

**Figure 2:**
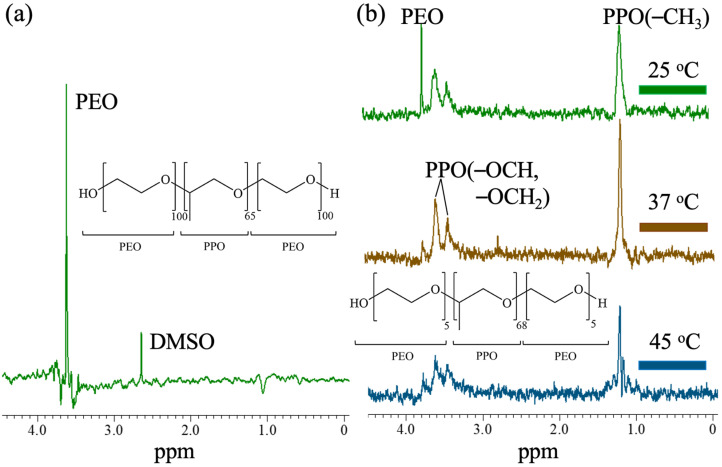
(**a**) HOESY of 5-FU embedded in F127 at 25 °C. (**b**) HOESY of 5-FU embedded in L121 at 25 °C, 37 °C, and 45 °C.

Figure 2 shows the measurement results of HOESY. Figure 2a demonstrates the HOESY spectrum of Pluronic F127 mixed with 4 mM of the 5-FU medicine. The DMSO signal at 2.7 ppm and the PEO signal at 3.7 ppm derived from the 5-FU medicine and Pluronic F127, respectively, can be clearly seen in the spectrum. This spectrum result shows that because Pluronic F127 has a longer hydrophilic PEO segment, at least three times that of the PPO segment, most of the 5-FU medicine is captured by the hydrophilic segment of Pluronic F127. Moreover, the DMSO spectrum signal is strong and obvious because the initial configuration conditions of the 5-FU medicine are dissolved in DMSO. This indicates that the 5-FU medicine was surrounded by the DMSO solvent and then eventually distributed among the hydrophilic PEO segments of Pluronic F127. In Figure 2b, the HOESY spectrum of Pluronic L121 mixed with 4 mM of the 5-FU medicine is shown. It can be clearly seen that the 5-FU medicine moved between the hydrophilic and hydrophobic segments of Pluronic L121. At 25 °C, the energy transferred through the fluorine atom resonance could reach the hydrophilic and hydrophobic segments of Pluronic L121, which are, respectively, displayed at 3.7 ppm and 3.5, 3.4, and 1.1 ppm, which are estimated to be evenly distributed. When the temperature reached 37 °C and 45 °C, the signal of the PEO segment at 3.7 ppm almost disappeared, and the signal of the PPO segment at 1.1 ppm was relatively enhanced, confirming that most of the 5-FU medicine was concentrated in the hydrophobic PPO segment. It is speculated that the 5-FU medicine prefers Pluronic F127 in the hydrophilic segment and Pluronic L121 in the hydrophobic segment. In order to produce a regulated drug release carrier, the combination of the two will have excellent drug release control capabilities.

**Figure 3:**
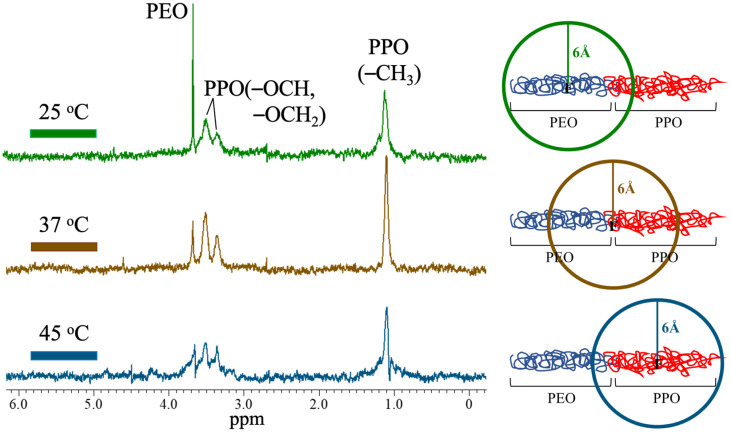
(**Left**): HOESY of the Pluronic blending composites with volume ratio of F127/L121 = 1/3 at 25 °C, 37 °C, and 45 °C. (**Right**): Schematic illusion of the molecular chain model in the spectra of HOESY.

The same measurement protocols of HOESY are reported in Figure 3, in order to observe the distribution of the 5-FU medicine in the F127/L121 Pluronic composites at 25 °C, 37 °C, and 45 °C. At 25 °C, although the F127/L121 Pluronic composite is already in the micelle state, the 5-FU medicine is distributed in both the hydrophilic and hydrophobic segments. When the temperature was raised to 37 °C, the hydrophilic PEO segment and the hydrophobic PPO segment signals can both be observed within the resonance energy transfer range of 6 Å of the fluorine atom. The difference is the deteriorated signal of the PEO segment at 3.7 ppm. Among them, for the measurement performed at 45 °C, the 5-FU medicine was mainly at the hydrophobic PPO segment, except for a barely observable signal from the PEO segment that was detected due to the signal around the copolymer junction point. Previous studies demonstrated that the amphiphilic nature of the Pluronic block copolymers would self-assemble into micellization in aqueous solutions above the critical micelle concentration (CMC) and the critical micelle temperature (CMT) [11,29]. Obviously, the stable micellization at a fixed concentration occurs above the CMT, and it becomes a driving force for the migration of the 5-FU medicine leaving away from the hydrophilic PEO segment in the self-assembly. Despite the fact the HOESY experiments were performed at ascending temperatures, the kinetics for the migration of the 5-FU medicine or the reversible process in short-term and long-term stability issues, which influence their shelf life, handling, and storage conditions, should be investigated in the future. The established 5-FU/F127/L121 Pluronic blending composites have a micelle structure that acts as a microenvironment mimicking the cell function to uptake or expel the 5-FU medicine by an external stimulus.

**Figure 4:**
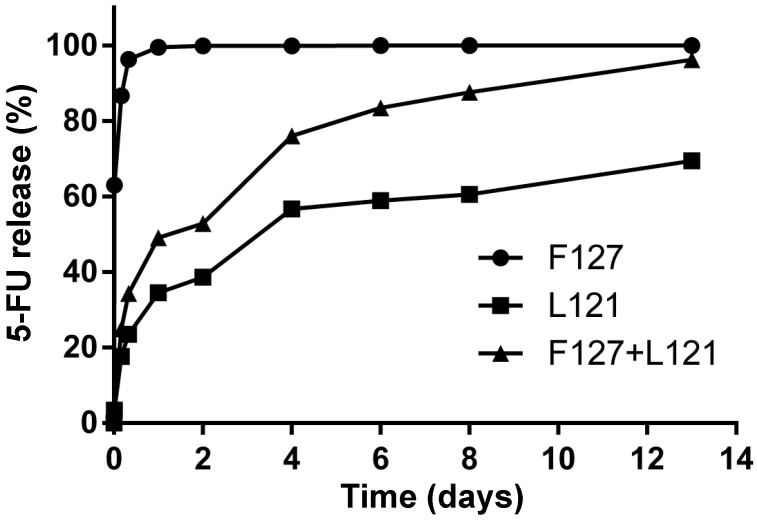
Curves of 5-FU drug release were plotted for F127, L121, and 5-FU/F127/L121 Pluronic blending composites at 37 °C.

In Figure 4, the curves of 5-FU drug release are plotted for the 5-FU/Pluronic F127, 5-FU/Pluronic L121, and 5-FU/F127/L121 Pluronic blending composites. The drug release from polymeric systems can be described by a semi-empirical equation, the Korsmeyer-Peppas model, which has a power-law relationship:

$$\frac{M_{t}}{M_{\infty }}=Kt^{n}$$

where *M_t_* and *M_∞_* are the absolute cumulative amounts of drug released at time *t* and infinite time, respectively; *K* is the release constant; exponent *n* describes the kinetic and the release mechanism [30,31,32,33,34]. The calculated *n* values are 0.05, 0.31, and 0.31 with respect to Pluronic F127, Pluronic L121, and F127/L121. This well represents the idea that the drug release mechanism of 5-FU followed Fick’s laws of diffusion since the value is below 0.43. In contrast, the release constant K determines the drug release rate accordingly at 0.93, 0.33, and 0.46. As expected, Pluronic F127 has a really fast drug release rate of less than one day, resulting from the 5-FU medicine loading at the hydrophilic PEO segments. The 5-FU medicine was directly released from the hydrophilic corona without any molecular resistance. Meanwhile, Pluronic L121 has a pretty slow drug release rate, and it took longer than twelve days until the 5-FU drug loading was completely released from the hydrophobic core of the micelles. Thus, a regulated drug release carrier was presented for the polymer blending of Pluronic F127 and Pluronic L121 in the volume ratio of 1:3. The curve of 5-FU drug release of the 5-FU/F127/L121 Pluronic blending composites steadily ascended within two weeks. This result tells us that the 5-FU/F127/L121 Pluronic blending composites would work effectively by slowing or stopping cancer cell growth in a designated medicine concertation after the first deployment surrounding the tumor site.

Finally, these HOESY and drug release studies suggest that Pluronic^®^ polymers have a broad amphiphilic nature in response to biological activities. Based on the better understanding of the 5-FU medicine loading and the migration in the Pluronic micelles, it has gradually become clear that this medical treatment can be applied in the tumor microenvironment [35,36]. This unique drug delivery system is one of the most remarkable impacts in the emergent field of nanomedicine.

## 4. Conclusions

This NMR study demonstrates that HOESY spectroscopy can be applied for the structural characterization of 5-FU drug-loaded Pluronic^®^ polymers, considering their potency as polymer-based drug delivery precision medicines. The results show that the distribution of the 5-FU medicine in the Pluronic micelles will move across the interface between the hydrophilic corona and the hydrophobic core according to the external temperature. The drug loading location of the 5-FU medicine is dominated by PEO segments at 25 °C. As the temperature increases from 25 to 45 °C, the 5-FU medicine migrates aggressively to the hydrophobic core inside the micelles. The 5-FU medicine was embedded into the PPO segment of Pluronic^®^ polymers above 37 °C due to stable micellization. The precise 5-FU loading in the hydrophobic area gives rise to prolonged and sustained drug release in daily clinical practice. Thus, the drug loading location and drug-carrier interactions are able to be designed to deliver drugs at predetermined release rates. The drug-carrier formulation developed here would diminish the side effects and improve the life quality of patients. The methodology could also apply to another chemotherapeutic medicine, gemcitabine, as well as to the fluorine atom content.

## Data Availability

Not applicable.
